# Acute Myocarditis Induced by Hepatitis E: An Uncommon Association

**DOI:** 10.1016/j.cjco.2022.04.008

**Published:** 2022-04-30

**Authors:** Héloïse Emeriaud, Fabien Huet, François Roubille, Jean-Luc Pasquié

**Affiliations:** a Department of Cardiology, Montpellier University Hospital, Montpellier, France; b PhyMedExp, Université de Montpellier, INSERM, CNRS, Cardiology Department, CHU de Montpellier, Montpellier, France; c Department of Cardiology, Vannes Regional Hospital, Vannes, France

## Abstract

Acute myocarditis is often caused by viral infections. Hepatitis E infection inflicts over 20 million people worldwide each year. Common extra-hepatic manifestations of hepatitis E infection include neurologic, hematologic, and renal sequelae.^1^ Acute myocarditis, defined by the presence of myocardial inflammatory infiltrates associated with nonischemic myocytic necrosis, is uncommon. Published reports of such cases are limited, and here we present the case of a 45-year-old man with acute myocarditis from hepatitis E infection. This case is the first described in Europe of acute myocarditis associated with hepatitis E infection.

## Résumé

La myocardite aiguë est souvent d'origine virale. Chaque année, plus de 20 millions de personnes dans le monde contractent l’hépatite E. Les manifestations extrahépatiques courantes de l’hépatite E comprennent des atteintes neurologiques, hématologiques et rénales. La myocardite aiguë, définie par la présence d’infiltrats inflammatoires myocardiques associés à une nécrose myocytaire non ischémique, est rare. Peu de cas ont été rapportés dans la littérature médicale. Nous abordons ici le cas d’un homme de 45 ans présentant une myocardite aiguë liée à une infection par le virus de l’hépatite E. Il s’agit du premier cas de ce genre décrit en Europe.

Viral myocarditis is a major cause of morbidity and mortality worldwide. The known profile of viruses involved has changed over the years. Although hepatitis B and C are well-known causes of myocarditis, hepatitis E as a cause of myocarditis rarely has been reported. Here, we describe a case of acute myocarditis secondary to hepatitis E infection.

## Case

A previously healthy 45-year-old man presented to the emergency room with a 2-day history of acute retrosternal chest pain associated with a burning sensation. He denied taking any prescription or illicit drugs. His only cardiovascular risk factor was a 23-pack-year history of active smoking. On review of systems, he reported no dyspnea, palpitations, fever, or abdominal symptoms. He had not recently travelled or received any blood product transfusion, but he endorsed regular consumption of cooked meats and sausages.

**Figure 1:**
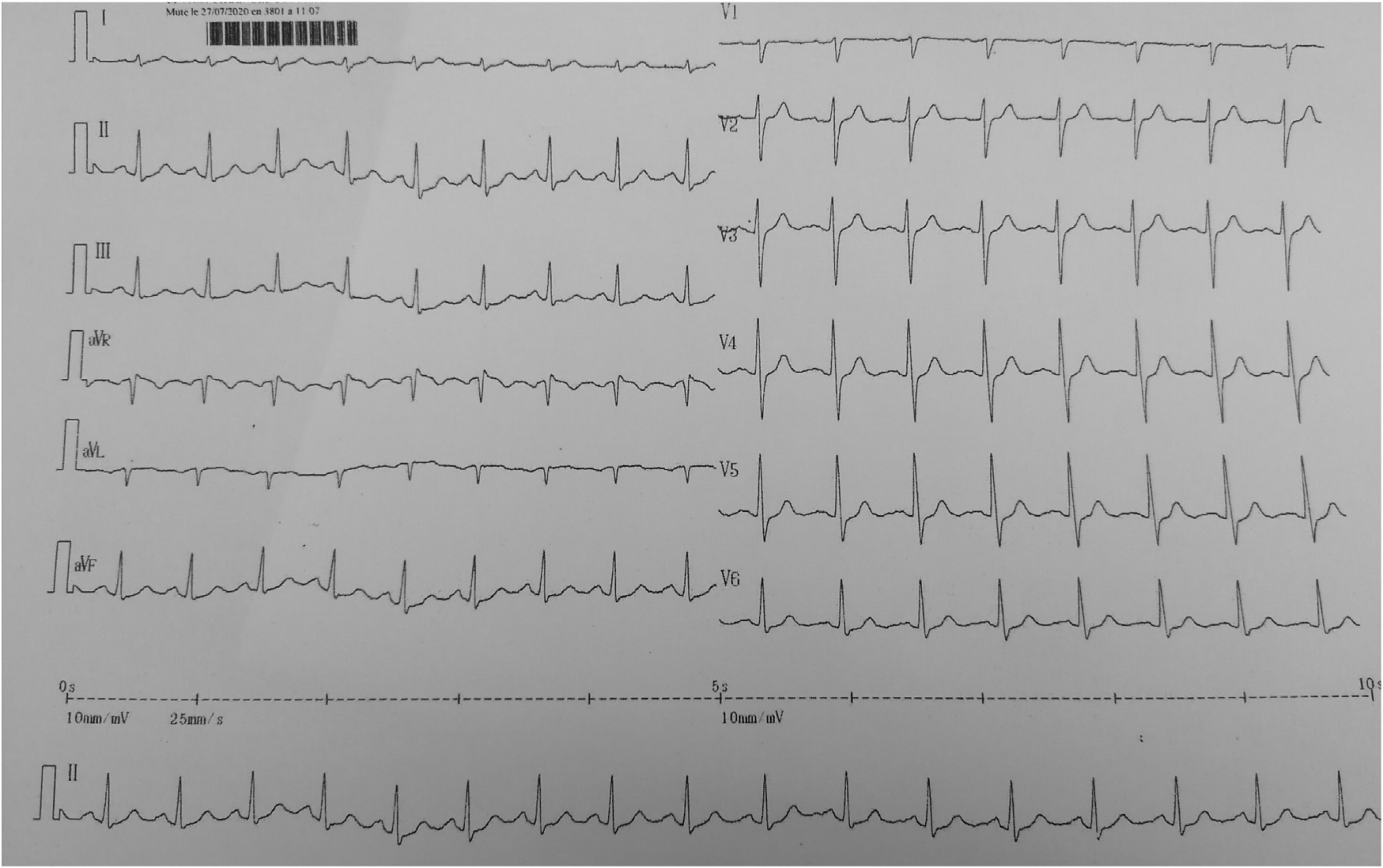
Electrocardiogram showing sinus rhythm with inferolateral millimetric ST depression.

**Figure 2:**
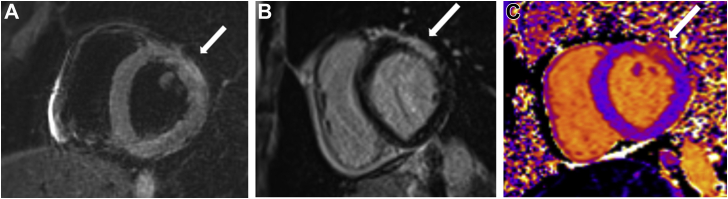
Cardiac magnetic resonance imaging showing (**A**) a spontaneous hypersignal T2 in the anterior and lateral territory in favour of edema, (**B**) anterolateral subepicardial enhancement on late gadolinium enhancement imaging, and (**C**) T1 mapping image in the same territory suggesting cardiomyocytic necrosis.

Results of his physical exam were within normal limits. Specifically, his precordial exam was normal, and his abdomen was soft and non-tender without hepatosplenomegaly. A chest radiograph was normal. A transthoracic echocardiogram showed preserved left ventricular ejection fraction (65%), inferobasal wall hypokinesia, normal right ventricular function, and no pericardial effusion. However, the electrocardiogram showed inferolateral ST depression (Fig. 1) in the setting of elevated levels of cardiac biomarkers (troponin, 782 ng/L; creatinine phosphokinase, 292 UI/L). Other bloodwork results (namely of liver-function tests and C-reactive protein level) and the coronary angiogram were normal. This constellation of findings raised a strong suspicion of myocarditis. Cardiac magnetic resonance imaging noted anterior and lateral patchy subepicardial contrast enhancement without subendocardial abnormalities associated with T1 hyperintensity, suggesting fibrosis development (Fig. 2)

The evolution was favourable, without recurrence of chest pain. We noticed hepatic cytolysis (aspartate aminotransferase, 105, ie, 2 ULN; alanine aminotransferase, 145, ie, 3 ULN; reference ranges: < 40, and < 41, respectively) without cholestasis 8 days after the onset of symptoms. The transthoracic echocardiogram showed complete recovery on day 10.

A complete etiologic assessment was carried out 2 days after admission and found seroconversion of hepatitis E (positive anti hepatitis E virus (HEV) IgM and IgG; negative HEV RNA). No co-infection with hepatitis B or C virus, human immunodeficiency virus (HIV), varicella-zoster virus (VZV), or human herpesvirus 6 (HHV6) was present; parvovirus B19 serologies were negative. The autoimmune balance, including anti-neutrophil cytoplasmic autoantibody (ANCA), antinuclear anitbodies, rheumatoid factor, serum complement titer, and immunoglobulin assay, was normal. The patient was discharged on day 11 after admission, when he was totally asymptomatic. His treatment included colchicine, 1 mg/d, for 3 months with bisoprolol, 1.25 mg/d. He was discharged on a beta blocker to avoid ventricular hyperexcitability caused by the inflammation of the myocardium.

## Discussion

Viral infections caused by cardiotropic viruses are very common in infectious acute myocarditis (eg, Coxsackie virus, enterovirus, influenza virus, parvovirus B19, and adenovirus; Supplemental Fig. S1).

The gold standard for the diagnosis of acute myocarditis is based on endomyocardial biopsy using the Dallas criteria. Endomyocardial biopsy is rarely performed in common practice, except in fulminant cases. Current guidelines recommend it more specifically in severe forms, including cardiogenic shock and ventricular systolic dysfunction associated with ventricular rhythm or conduction disorders, and in patients who do not improve after a few days despite conventional supportive therapy. The diagnosis is therefore based on electrocardiogram, biological assessment, echocardiography, magnetic resonance imaging, and exclusion of coronary artery disease.^2^

Cardiac magnetic resonance imaging has become mandatory for myocarditis management. According to the 2018 Lake Louise criteria, 2 criteria are indicative of active myocarditis. The criteria are regional or global myocardial edema highlighted by T2-weighted sequence hypersignal and myocardial necrosis or fibrosis, most often multifocal epicardial localization demonstrated by late enhancement to gadolinium in T1 weighted sequence. These criteria are based on at least one T1-based criterion (increased myocardial T1 relaxation times, extracellular volume fraction, or late gadolinium enhancement) plus at least one T2-based criterion (increased myocardial T2 relaxation times, visible myocardial edema, or an increased T2 signal intensity ratio).^3^^,^^4^

Acute myocarditis is uncommon in hepatitis E infection. In emerging countries, strictly human genotype 1 and 2 viruses are transmitted via the feco-oral route (ingestion of contaminated water). In developed countries, HEV is transmitted by food (mainly undercooked pork). HEV is actually the leading cause of fulminant hepatitis worldwide. Biological diagnosis is based on the detection of IgM antibodies against HEV. The diagnosis may also be reached by the presence of IgG HEV antibodies or positive results of a polymerase chain reaction test in blood and stool samples.^5^

In the cases reported in India, patients were younger (between 22 and 26 years old), with an initial clinical presentation of jaundice and decreased urine output. In comparison to our current case, the heart damage appears to be more severe, with global hypokinesia and severe dysfunction of the left ventricle seen on the echocardiograms. Cardiac magnetic resonance imaging was performed on 1 patient only. Among these 4 cases that occurred and were described in India, 1 patient died.^6^^,^^7^

The case of acute hepatitis E infection concomitant with the development of acute myocarditis presented here suggests that they have an etiologic link. However, other cardiotropic viruses, such as coxsackievirus and enteroviruses, have not been tested, which can be considered an important limitation, especially given that these pathogens are also transmitted by the fecal-oral route. Although endomyocardial biopsy was not performed (following our local consensus), we provide strong clues for its implication here. This clinical case shows that HEV infection can be complicated by acute myocarditis.

## Conclusion

The simultaneous occurrence of acute myocarditis with acute hepatitis E suggests an etiologic link between these 2 diseases. This association shows that benign and asymptomatic hepatitis E can be complicated by myocarditis. To our knowledge, fewer than 10 cases of this rare association have been reported. We thus believe that hepatitis E should be kept in mind as a possible viral etiology of acute myocarditis.

**Novel Teaching Points**

• Myocarditis is a major cause of morbidity and mortality worldwide.
• The main causes of myocarditis are viral infections.
• Reports of hepatitis E as a cause of myocarditis are rare.
• We describe here a case of acute myocarditis associated with hepatitis E infection.

## References

[1] Fousekis F.S., Mitselos I.V., Christodoulou D.K. (2020). Extrahepatic manifestations of hepatitis E virus: an overview. Clin Mol Hepatol.

[2] Caforio A.L.P., Pankuweit S., Arbustini E. (2013). Current state of knowledge on aetiology, diagnosis, management, and therapy of myocarditis: a position statement of the European Society of Cardiology Working Group on Myocardial and Pericardial Diseases. Eur Heart J.

[3] Friedrich M.G., Marcotte F. (2013). Cardiac magnetic resonance assessment of myocarditis. Circ Cardiovasc Imaging.

[4] Ferreira V.M., Schulz-Menger J., Holmvang G. (2018). Cardiovascular magnetic resonance in nonischemic myocardial inflammation: expert recommendations. J Am Coll Cardiol.

[5] Aggarwal R. (2013). Diagnosis of hepatitis E. Nat Rev Gastroenterol Hepatol.

[6] Sengupta P., Biswas S., Roy T. (2019). Hepatitis E-induced acute myocarditis in an elderly woman. Case Rep Gastroenterol.

[7] Premkumar M., Rangegowda D., Vashishtha C. (2015). Acute viral hepatitis E is associated with the development of myocarditis. Case Rep Hepatol.

