# Supplementary figures and images for: Acute Myocarditis Induced by Hepatitis E: An Uncommon Association
Source: CJC Open. 2022 Apr 30;4(8):729–31. doi: 10.1016/j.cjco.2022.04.008 (PMC9402964; doi:10.1016/j.cjco.2022.04.008)

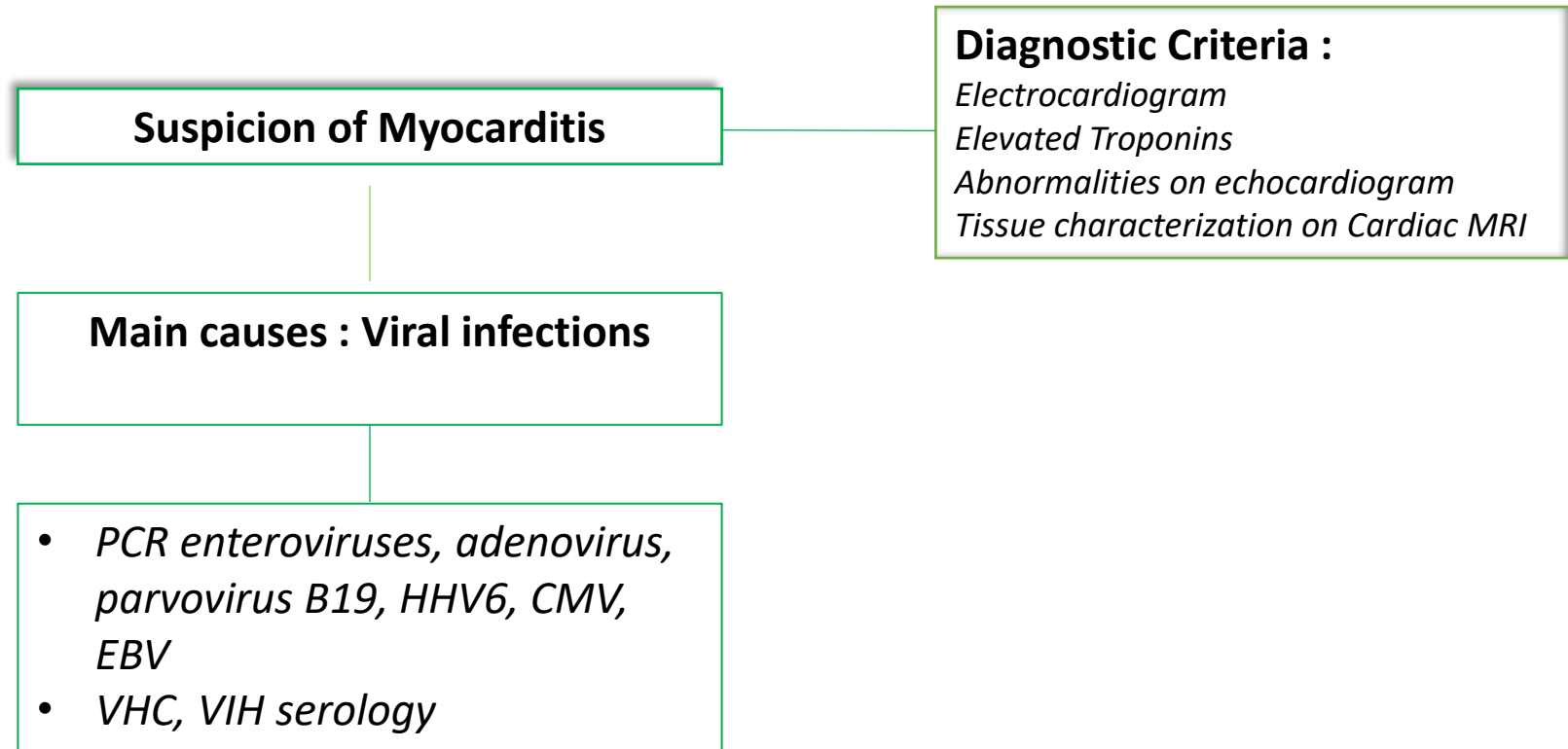

**Supplemental Figure S1.** Diagnostic algorithm for acute myocarditis

Supplement: Supplemental Figure S1 [file mmc1.pdf]
